# Effectiveness of Coenzyme Q10 Supplementation in Statin-Induced Myopathy: A Systematic Review

**DOI:** 10.7759/cureus.68316

**Published:** 2024-08-31

**Authors:** Khoula Ahmad, Naelijwa J Manongi, Ramkumar Rajapandian, Sajida Moti Wala, Esraa M Al Edani, Essa A Samuel, Ana P Arcia Franchini

**Affiliations:** 1 Internal Medicine, California Institute of Behavioral Neurosciences and Psychology, Fairfield, USA; 2 Family Medicine, California Institute of Behavioral Neurosciences and Psychology, Fairfield, USA; 3 Trauma and Orthopedics, California Institute of Behavioral Neurosciences and Psychology, Fairfield, USA; 4 Dermatology, California Institute of Behavioral Neurosciences and Psychology, Fairfield, USA; 5 Physical Medicine and Rehabilitation, California Institute of Behavioral Neurosciences and Psychology, Fairfield, USA; 6 Research, California Institute of Behavioral Neurosciences and Psychology, Fairfield, USA

**Keywords:** statins, hmg-coa reductase inhibitors, musculoskeletal disorder, ubiquinone, coenzyme q10 (coq10)

## Abstract

Statins are among the most widely prescribed drugs for treating dyslipidemia and reducing the incidence of heart disease and stroke. However, they come with a wide range of side effects, from myopathy to necrotizing rhabdomyolysis, as well as diabetes, hepatotoxicity, and sleep problems. The most common side effect of statins is statin-induced myopathy, often leading to discontinuation of statin therapy and noncompliance in many patients. This study aims to assess the effectiveness of coenzyme Q10 (CoQ10) supplementation as a treatment for patients with statin-induced myopathy. This systematic review was conducted by following the Preferred Reporting Items for Systematic Reviews and Meta-Analyses (PRISMA) 2020 statement. Relevant studies were identified through searches of Medline, PMC, PubMed, Science Direct, and Google Scholar. Only randomized control trials and meta-analyses of oral CoQ10 supplementation versus placebo in adults with statin-associated myalgia were included. The risk of bias was assessed using the Cochrane Risk of Bias tool (The Cochrane Collaboration, London, England, UK) and the measurement tool for the "assessment of multiple systematic reviews" (AMSTAR tool). Out of 5,000 records identified, only five were selected for this review: one meta-analysis and four randomized controlled trials. All of these studies were conducted between 2010 and 2023, involving a total of 800 patients. All randomized controlled trials showed improvement in statin-associated myopathy with CoQ10 supplementation, along with or without a reduced dosage of statins, without any notable side effects of CoQ10. Therefore, it can be deduced that CoQ10 supplementation significantly ameliorates statin-induced musculoskeletal symptoms.

## Introduction and background

In the United States, two in five adults suffer from dyslipidemia or high cholesterol (total cholesterol >200 mg/dl) [1]. Dyslipidemia refers to the imbalance of lipids in the body and includes one or more of the following: increased low-density lipoprotein (LDL)-cholesterol level, increased total cholesterol level, increased triglyceride level, and decreased high-density lipoprotein (HDL)-cholesterol level [2]. Lipids, such as cholesterol or triglycerides, are absorbed from the intestines and transported throughout the body with the help of lipoproteins. These lipids are used for energy, steroid production, and bile acid synthesis. Determinants contributing to this process include cholesterol, LDL, triglycerides, and HDL. A disproportion in any of these factors, whether from organic or non-organic causes, can lead to dyslipidemia [3]. Dyslipidemia significantly contributes to atherosclerotic coronary artery disease and stroke, which are among the leading causes of death in the United States [4-12]. Appropriate treatment of dyslipidemia can result in reduced heart-related mortality as well as overall mortality [5,6,13]. The incidence of dyslipidemia increases with age. One of the most effective pharmacological treatments for dyslipidemia is the use of 3-hydroxy-3-methylglutaryl coenzyme A (HMG-CoA) reductase inhibitors, also known as statins, which block the production of products like cholesterol, dolichol, geranyl-pyrophosphate, farnesyl-PP, and ubiquinone or coenzyme Q10 (CoQ10) by inhibiting the conversion of HMG-CoA to mevalonate. Statins reduce LDL-cholesterol on average by 30-50% [14]. Furthermore, statins can also modify endothelial function and the inflammatory process caused by atherosclerotic plaque while inhibiting smooth muscle proliferation, thus playing an important role in the primary and secondary prevention of heart diseases [15,16]. Although the use of statins decreases cholesterol production, which helps with reducing dyslipidemia, it has various side effects ranging from myalgia (muscle pain/weakness) to rhabdomyolysis (muscle tissue breakdown), as well as hepatotoxicity, sleep disturbance, and memory loss.

Myopathy is the most common side effect of statin treatment, with the incidence of statin-induced myopathy in patients receiving statins being 27.8%. However, this may vary depending on the dosage of statin, the type of statin prescribed, the biological gender of the patient, genetic predisposition, baseline health of the patient, and coinciding comorbidities. However, the exact mechanism behind statin-induced myopathy is not yet identified. One common theory is the deficiency of CoQ10 caused by statins. This is because CoQ10 is a part of the mitochondrial membrane and is involved in the electron transport chain and oxidative phosphorylation. Its deficiency results in impaired vitamin E production and muscle energy metabolism. Other causes include oxidative stress, protein prenylation, cellular apoptosis, and impaired insulin receptor pathway [17].

Around one in five statin users stops using statins for 12 months or more, and the largest reason among statin-related causes is myopathy, followed by musculoskeletal disorders [18]. One of the commonly proposed ideas to curb statin-related myopathy is the supplementation of CoQ10 to mitigate myopathic symptoms. The purpose of this paper is to investigate the efficacy of CoQ10 concerning curbing or reducing the effects of statin-related myopathy.

## Review

Methodology

This systematic review follows the guidelines and principles of the Preferred Reporting Items for Systematic Reviews and Meta-Analyses (PRISMA) 2020 [19].

Search Strategy

Search engines used were PubMed, Medline, PMC, and Science Direct. Statins, myopathy, and CoQ10 were three basic concepts in the research question. Boolean term “OR” was used to connect different concepts and keywords. Using these concepts and keywords, Medical Subject Heading (MeSH) as given below was created to use in PubMed Advanced Search: myopathy OR muscle pain OR skeletal muscle disorder OR polymyositis OR skeletal muscle inflammation OR myotoxicity OR mitochondrial myopathy OR muscle wasting OR ("myositis/complications"[Mesh] OR "myositis/diet therapy"[Mesh] OR "myositis/drug therapy"[Mesh] OR "myositis/enzymology"[Mesh] OR "myositis/pathology"[Mesh] OR "myositis/physiopathology[Mesh] OR "myositis/therapy"[Mesh])) AND (statins OR LDL-lowering drugs OR cholesterol-lowering drugs OR HMG-CoA reductase inhibitors OR atorvastatin OR fluvastatin OR lovastatin OR pitavastatin OR pravastatin OR rosuvastatin OR simvastatin OR (hHydroxymethylglutaryl-CoA reductase inhibitors/adverse effects"[Mesh] OR "hydroxymethylglutaryl-CoA reductase inhibitors/metabolism"[Mesh] OR "hydroxymethylglutaryl-CoA reductase inhibitors/pharmacokinetics"[Mesh] OR "hydroxymethylglutaryl-CoA reductase inhibitors/pharmacology"[Mesh] OR "hydroxymethylglutaryl-CoA reductase inhibitors/therapeutic use"[Mesh] OR "hydroxymethylglutaryl-CoA reductase inhibitors/toxicity"[Mesh]))) AND (coenzyme Q OR CoQ10 OR ubiquinone OR mitoquinone OR ubidecarenone OR ubiquinone OR vitamin Q10 OR ("ubiquinone/deficiency"[Mesh] OR "ubiquinone/drug effects"[Mesh] OR "ubiquinone/metabolism"[Mesh] OR "ubiquinone/pharmacokinetics"[Mesh] OR "ubiquinone/pharmacology"[Mesh] OR "ubiquinone/physiology"[Mesh] OR "ubiquinone/therapeutic use"[Mesh].

Article Selection and Screening

Articles were selected using the specified keywords and MeSH strategy in the aforementioned search engines. Relevant articles underwent rigorous screening based on their title, abstract, and subsequent reading of the full articles after removing duplicates. Subsequently, five papers were finalized and selected for quality appraisal using quality appraisal tools.

Inclusion Criteria

Only review articles, such as systematic review meta-analyses and randomized controlled trials, were selected that were in English and published within the last 14 years (2010-2024). We also made sure all the studies should only involve humans and are easily available as free full-text articles. We also selected articles about statin users and various side effects they developed to result in non-compliance, and we focused on articles that include the use of CoQ10 in statin users.

Exclusion Criteria

Articles dated before 2010 and not primarily in English were excluded. Additionally, non-randomized controlled trials, case studies, in vitro studies, animal studies, and grey literature were also excluded. Studies involving non-statin users were also excluded. Articles discussing CoQ10 supplementation in muscle disorders unrelated to statins were also excluded.

Quality Appraisal

Finalized articles were systematic reviews, meta-analyses, and randomized control trials; hence, quality appraisal tools used were the measurement tool for the "assessment of multiple systematic reviews" (AMSTAR) [20] for systematic reviews and meta-analyses and the Cochrane Risk of Bias tool (The Cochrane Collaboration, London, England, UK) [21] for randomized control trials, as mentioned in Table 1 and Table 2, respectively.

**Table 1:** Quality appraisal of review articles using the AMSTAR checklist AMSTAR: measurement tool for the "assessment of multiple systematic reviews"

Study characteristics	Qu et al. [22]
1. Was an "a priori" design provided (the research question and inclusion criteria should be established before the review)?	Yes
2. Was there duplicate study selection and data extraction?	Yes
3. Was the comprehensive literature search performed?	Yes
4. Was the publication status used as an inclusion criterion?	Yes
5. Was the list of studies (included or excluded) provided?	Yes
6. Are the characteristics of included studies provided?	Yes
7. Was the scientific quality of the included studies assessed and documented?	Yes
8. Was the scientific quality of included studies used appropriately in formulation conclusions?	Yes
9. Were methods used to combine findings of studies appropriate?	Yes
10. Was the likelihood of publication bias assessed?	Yes
11. Was the conflict of interest stated?	Yes

**Table 2:** Quality appraisal of randomized controlled trials

Study characteristics	Derosa et al. [23]	Skarlovnik et al. [24]	Yasser et al. [25]	Zlatohlavek et al. [26]
1. Random sequence generation (selection bias)	Yes	Yes	Yes	Yes
2. Allocation concealment (selection bias)	Yes	Yes	Yes	Yes
3. Blinding of personnel and participants	Yes	Yes	Yes	Yes
4. Blinding of outcome assessment	Yes	Yes	Yes	Yes
5. Incomplete outcome data	Yes	Yes	Yes	Yes
6. Selection report	No	Yes	Yes	Yes
7. Other bias	No	No	Yes	Yes

Result

**Figure 1:**
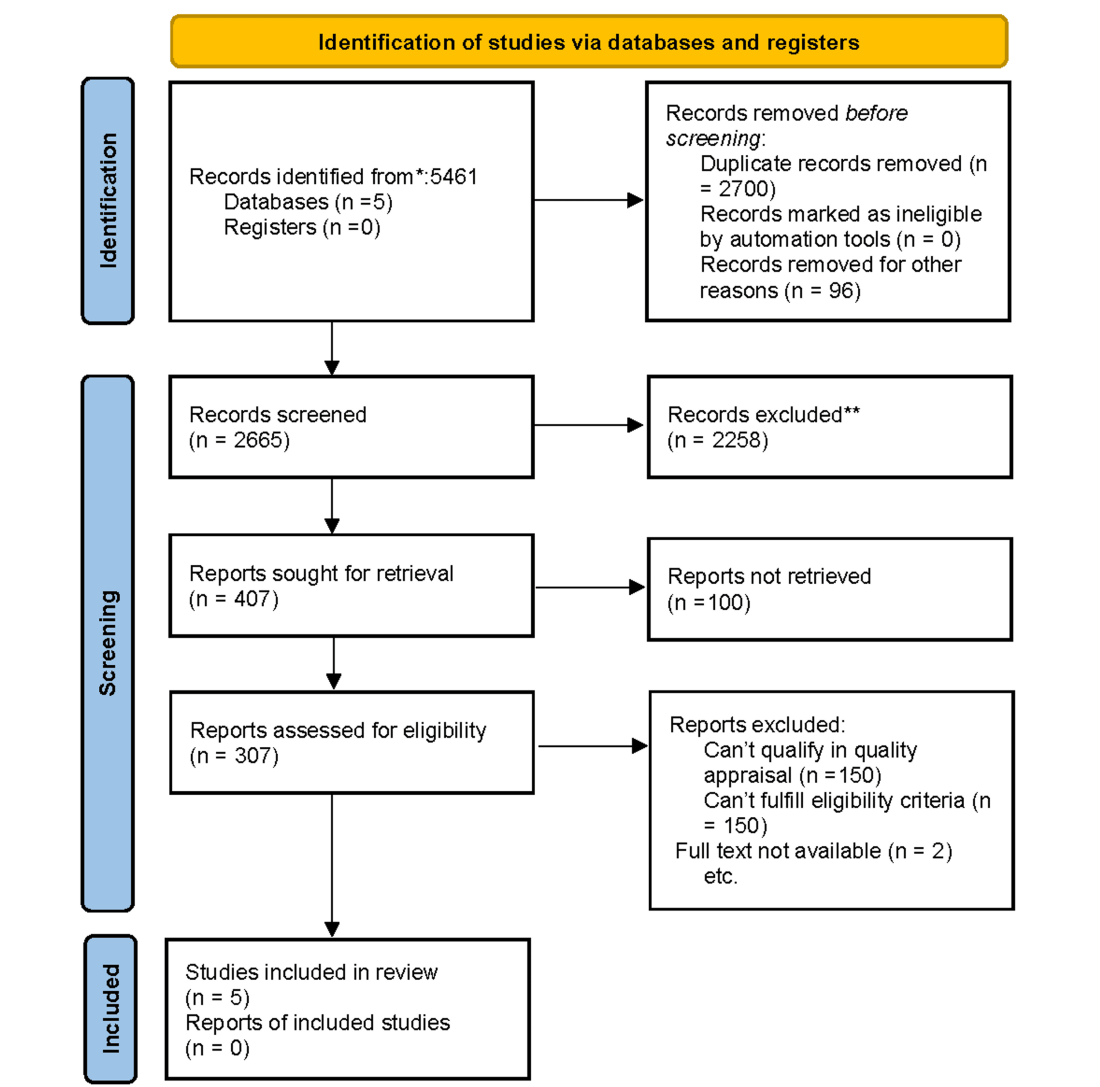
PRISMA 2020 flowchart depicting the process for article selection * Consider, if feasible, reporting the number of records identified from each database or register searched (rather than the total number across all databases/registers). ** If automation tools were used, indicate how many records were excluded by humans and how many were excluded by automation tools. PRISMA: Preferred Reporting Items for Systematic Reviews and Meta-Analyses

A total of 5,461 relevant articles were identified using PubMed, PMC, Medline, Google Scholar, and Science Direct. After removing duplicates and applying inclusion criteria, only 307 articles remained. We then shortlisted a total of 36 articles by reviewing titles and abstracts. From the shortlisted articles, those that did not meet our eligibility criteria or for which the full text could not be retrieved were removed, leaving five articles finalized for review. The selection process of the studies is depicted in Figure 1 in the PRISMA flowchart.

Our primary question was to study the effect of CoQ10 supplementation in statin-associated myopathy, with the secondary motive of studying statins and skeletal muscle CoQ10 levels. A summary of the articles included is mentioned in Table 3.

**Table 3:** Summary of studies included in this article CoQ10: coenzyme Q10, RCT: randomized controlled trial, CPK: creatine phosphokinase, SAMS: statin-associated muscular symptoms, HDL: high-density lipoprotein

Author, title, and year of publication	Study design	Group sample size	Intervention studied	Result	Conclusion
Qu et al. (2018); Effects of coenzyme Q10 on statin-induced myopathy: an updated meta-analysis of randomized controlled trial [22]	Meta-analysis of RCTs	575	To assess the effect of CoQ10 supplementation on statin-induced myopathy	294 statin users with statin-induced muscular symptoms were given CoQ10 supplements and 281 of the total patients were enrolled in a placebo group and their muscular symptoms were assessed. The treatment group showed significant improvement without any side effects	CoQ10 reduces statin-induced myopathy significantly
Derosa et al. (2019); Coenzyme Q10 liquid supplementation in dyslipidemic subjects with statin-related clinical symptoms: a double-blind, randomized, placebo-controlled study randomized controlled trial [23]	RCT	60	Effect of CoQ10 supplementation of 100 mg with reduced statin dose on statin-induced myopathy	The Clinical Index Score for myalgia was improved after 3 months with CoQ10, while no change was observed in the placebo	The supplementation of CoQ10 with half dosage statin in previously statin-intolerant patients improves clinical symptoms such as myalgia or pain
Skarlovnik et al. (2014): Coenzyme Q10 supplementation decreases statin-related mild-to-moderate muscle symptoms: a randomized clinical study [24]	RCT	50	Effect of CoQ10 supplementation 50 mg twice daily on statin-induced muscular complaints	25 patients were given 50 mg CoQ10 twice daily and 25 patients were given a placebo tablet and both groups were assessed after 30 days. Pain severity score and pain interference score improved in the treatment group	CoQ10 helps ameliorate statin-induced muscular complaints
Yasser et al. (2021); Assessment of some clinical and biochemical parameters after combining coenzyme Q10 to statin in dyslipidemic patients [25]	RCT	52	CoQ10 200 mg was given to patients in the treatment group along with the atorvastatin vs placebo group in which only atorvastatin was given, and the effect was observed after 12 weeks for CPK, lipid peroxidation, SAMS, lipid profile, liver enzymes, and metabolic parameters were assessed	Patients in the treatment group showed improvement in muscular symptoms as well as raised serum CoQ10 levels along with a reduction in CPK and lipid peroxidation while improving HDL levels	Signify the role of CoQ10 as a potential treatment of statin-induced myopathy
Zlatohlavek et al. (2012): The effect of coenzyme Q10 in statin myopathy [26]	RCT	28	To study the effect of CoQ10 supplementation in reducing statin-associated myopathy	28 patients were given statins of various doses and types, and when they reported muscular complaints. CoQ10 supplement was given. They were then assessed over a period of 3 to 6 months and it was established that CoQ10 is effective in reducing muscular complaints	CoQ10 is effective in statin-induced myopathy with side effects

Discussion

The incidence of dyslipidemia is continuing to rise due to trends such as consumption of processed food, tobacco use, sedentary lifestyles, obesity, and diets deficient in fruits, vegetables, and nuts. These trends are major contributing factors to dyslipidemia aside from genetic predisposition [2]. Dyslipidemia serves as the root cause of diseases such as stroke, carotid artery stenosis, coronary artery diseases, and mesenteric ischemia and can even lead to sudden cardiac arrest. The initial approach to treating dyslipidemia should involve dietary modifications, such as consuming diets rich in nuts, fruits, vegetables, and omega-3 fatty acids, tailored according to personalized caloric requirements, along with engaging in physical activity for at least 40 minutes, which can include activities such as brisk walking, three to five days a week.

Statins, also known as HMG-CoA reductase inhibitors, are the first line of medication for treating dyslipidemia prescribed after dietary modification and increased physical activity [2]. Statins inhibit cholesterol synthesis in the liver by competitively inhibiting HMG-CoA reductase, thus preventing the conversion of HMG-CoA to mevalonate. Mevalonate is an intermediate compound in the synthesis of cholesterol via squalene. Furthermore, mevalonate is converted into farnesyl pyrophosphate, a compound that gives rise to prenylated proteins that play a pivotal role in energy production and brain function [27]. Besides reducing cholesterol production in the liver, statins also inhibit smooth muscle proliferation, thereby stabilizing atherosclerotic plaque [15,16]. Statins play an undeniable role in decreasing the incidence of heart disease, stroke, and overall mortality [5,6,13]. Statins, while inhibiting cholesterol synthesis in the liver, also inhibit CoQ10's endogenous production, as mevalonate gives rise to farnesyl pyrophosphate, which is an intermediate in ubiquinone synthesis.

CoQ10, also known as ubiquinone, is a naturally occurring fat-soluble quinone found on the hydrophobic side of cell membranes. It is synthesized endogenously from the prenylation of geranylgeranyl pyrophosphate, which is initially derived from mevalonate during endogenous cholesterol synthesis or can be obtained from dietary fat. CoQ10 is a redox-active benzoquinone ring conjugated to an isoprenoid chain, the length of which differs among species. In humans, ubiquinone primarily contains 10 isoprenyl units and is designated as CoQ10. It is a component of the electron transport chain in the inner mitochondrial matrix, hence playing a crucial role in oxidative phosphorylation and energy production. Furthermore, ubiquinone also shields against damage by free radicals by producing active forms of antioxidants such as ascorbic acid and tocopherol (vitamin E) [27]. Deficiencies in CoQ10 can lead to respiratory chain defects, reactive oxygen species (ROS) production, and apoptosis, resulting in conditions such as encephalomyopathy, severe infantile multisystemic disease, cerebellar ataxia, isolated myopathy, and nephrotic syndrome.

On the other hand, statin intolerance or discontinuance or noncompliance by patients due to its intolerable adverse effects is also a reality. The elevation of transaminase enzymes in the liver, new-onset diabetes, and statin-associated muscular symptoms are major side effects that result in statin intolerance [28,29]. Statin-associated muscle complaints include myalgia, myopathy, and myositis with or without creatine kinase (CK) elevation, or in the most severe cases, rhabdomyolysis. Myalgia is an unexplained muscle discomfort without a rise in CK. Myopathy is defined as muscle weakness and tenderness with or without raised CK and rhabdomyolysis, which includes myonecrosis and myoglobinuria, resulting in acute kidney failure with high levels of CK [30]. The statin-associated muscle symptom clinical index, developed by the Muscle Safety Expert Panel, is a tool to identify symptoms that were reproducible on statin discontinuation and recurred on statin re-administration [30-32]. Higher scores depict the need to reduce the dose of previously administered statin or start another statin with the same pharmacodynamic effects or co-administration of another lipid-lowering drug. Scores on the lower end emphasize further cause evaluation of statin-associated muscular symptoms. Risk factors for statin-associated muscular symptoms that lead to intolerance are Asian descent, a family history of muscle symptoms upon lipid-lowering treatments, untreated hypothyroidism, type 1 diabetes, chronic liver disease, sex (female being more affected), treatment for hypertension, age (>80 years), frailty, multi-system diseases, vitamin D deficiency, and excessive physical activities [30].

A meta-analysis of randomized controlled trials published in 2015 on serum and muscular CoQ10 concentration to statin treatments showed decreased CoQ10 levels in both serum and muscles following statin therapy [33]. Another observation study conducted in 2020 concluded that statin users had lower serum CoQ10 and HMG-CoA reductase levels associated with nerve conduction deficits, suggesting a role of CoQ10 in the occurrence of neurological adverse effects [34]. This gives rise to one of the hypotheses that CoQ10 deficiency induced by statins is responsible for statin-induced muscular symptoms. This forms the basis of our research topic, aiming to assess the role of CoQ10 supplementation with statins in improving complaints of statin-associated myopathy. After rigorous analysis and careful selection of articles meeting the criteria of quality appraisal tools (>70% criteria), we focused on articles showing CoQ10's association with myopathic complaints of statin users.

Qua et al. published an updated meta-analysis comprising 12 randomized control trials involving 575 patients, which also identified CoQ10 supplementation along with statins as a complementary approach to reduced statin-induced myopathic complaints, thereby improving statin intolerance [22]. A double-blinded randomized placebo-controlled trial recruited 60 Caucasian patients intolerant to statins. After one month of wash-out period, they were again prescribed half a dose of statin with 100 mg/dl of liquid CoQ10 supplementation over three months, resulting in improvement in myopathic complaints caused by statins [23]. A randomized control trial performed by Skarlovnik et al. recruited statin users with myopathic complaints and divided them into a treatment group and a placebo group. The treatment group was given a 50 mg CoQ10 supplement twice daily over three months. Patients receiving CoQ10 supplements improved in pain severity score and pain interference score with daily activities [24].

In 2021, another randomized controlled trial involving statin-intolerant patients due to muscle pain supplemented with CoQ10 for 12 weeks demonstrated steady improvement in the muscle symptoms after CoQ10 adjuvant therapy among the patients with statin-associated muscle symptoms. This resulted in increased patient adherence to statins along with decreasing muscle complaints [25]. Zlatohlavek et al. published a study in 2012 in which 28 patients of middle age were monitored (18 women and 10 men) and treated with different types and doses of statin. Muscle weakness and pain were monitored using a scale of one to 10, on which patients expressed the degree of their inconvenience. Examination of muscle problems was performed before administration of CoQ10 and after three and six months of dosing. Statistical analysis of facts and figures concluded the effectiveness of CoQ10 supplementation in significantly improving muscle pain caused by statins [26].

Thus, the theory of reduced synthesis and consequently reduced levels of CoQ10 in skeletal muscle with statin use is responsible for statin-associated musculoskeletal symptoms, and therefore, supplementation of CoQ10 in patients with statin-induced myopathies is the most plausible solution without any reported adverse effect. Although statins also inhibit the production of CoQ10, our body synthesizes only half of CoQ10 endogenously, with the rest obtained through diet. Exogenously obtained CoQ10 cannot compensate for deficiency induced by statins, resulting in reduced serum and muscular CoQ10 levels, which contribute to the inhibition of oxidative phosphorylation due to mitochondrial dysfunction [27]. Mitochondria are essential for maintaining cellular homeostasis and overall skeletal muscle health. They serve as the “powerhouses” of the cell, producing most of the body’s energy through oxidative phosphorylation. When mitochondria malfunction, it can lead to a series of pathophysiological changes. These changes contribute to skeletal muscle atrophy, including the generation of ROS, disturbances in mitochondrial dynamics (fission and fusion), decreased mitochondrial biogenesis, impaired regulation of autophagy and mitophagy, and apoptosis, resulting in complaints ranging from muscle pain to rhabdomyolysis [35]. This mitochondrial dysfunction and damage caused by free radicals can be significantly avoided by incorporating CoQ10 along with statin treatment, thereby improving the adherence of patients to statin therapy, which plays an inevitable role in the primary prevention of other morbid conditions.

Limitations

This systematic review has several limitations due to the standards we developed as filters to screen research papers. We only included free full-text papers published between 2010 and 2024 and included only humans as study subjects. We excluded papers involving experiments on animals and those in other languages. This review included only randomized controlled trials and other meta-analyses and review articles; thus, variation in sample size and statistical techniques could be a factor for bias. Another hindering factor is that in the included articles, the sample population was followed over varied intervals of time, and the type and dosage of prescribed statin were also not consistent. To reduce bias, sample size, duration, dosage, and type of prescribed statin should be standardized. Another limitation is that patients included in these studies vary in terms of age, race, and baseline health status, which also affects the development of statin-induced myopathy.

## Conclusions

Statin-induced myopathy is the most prevalent cause of stain intolerance. Various hypotheses were proposed to identify the pathophysiology of myopathy in statin users. One of the hypotheses points toward inhibition of CoQ10 synthesis due to statin and consequently mitochondrial dysfunction of skeletal muscles contributing toward musculoskeletal complaints. The theory gives rise to the importance of CoQ10 supplementation along with the prescribed dosage of statins to reduce myopathic complaints. Different studies included in this systematic review reflect that CoQ10 supplementation considerably improved statin-associated myopathy, hence strengthening the hypothesis of the role of CoQ10 in statin-associated myopathy, as CoQ10 obtained from exogenous sources is not enough to alleviate statin-induced deficiency. Inhibition of oxidative phosphorylation, mitochondrial dysfunction, and secondary deficiency of antioxidants such as vitamin E and ascorbic acid cumulatively cause muscle membrane damage, resulting in a wide variety of complaints, from muscle pain to rhabdomyolysis with elevated creatinine kinase level, which is only prevented by CoQ10 supplementation along with statins.
